# Current Review of Optical Neural Interfaces for Clinical Applications

**DOI:** 10.3390/mi12080925

**Published:** 2021-08-02

**Authors:** Younghoon Park, Sung-Yun Park, Kyungsik Eom

**Affiliations:** Department of Electronics Engineering, College of Engineering, Pusan National University, Busan 46241, Korea; yhunp93@gmail.com

**Keywords:** optical neural interface, optical neuromodulation, optical functional imaging, clinical application

## Abstract

Neural interfaces, which enable the recording and stimulation of living neurons, have emerged as valuable tools in understanding the brain in health and disease, as well as serving as neural prostheses. While neural interfaces are typically based on electrical transduction, alternative energy modalities have been explored to create safe and effective approaches. Among these approaches, optical methods of linking neurons to the outside world have gained attention because light offers high spatial selectivity and decreased invasiveness. Here, we review the current state-of-art of optical neural interfaces and their clinical applications. Optical neural interfaces can be categorized into optical control and optical readout, each of which can be divided into intrinsic and extrinsic approaches. We discuss the advantages and disadvantages of each of these methods and offer a comparison of relative performance. Future directions, including their clinical opportunities, are discussed with regard to the optical properties of biological tissue.

## 1. Introduction

There are billions of neurons in the human body which are connected to organs for regulating their functions. As communication signals, action potentials are carried throughout the neurons to and from other organs to regulate their function. Due to these features, there has been growing interest in treating diseases by neurostimulation to regulate body functions [1]. In fact, there are numerous neural prosthesis devices (i.e., cochlear implant [2], retinal implant [3]) already developed to help patients suffering from diseases that have been known to be incurable by modern pharmacological or surgical treatment.

One of the key components of neural prostheses is the neural interface which bridges between the device and the nervous system [4,5]. Interfacing with the neural circuit allows us to read and control their behaviors. With regards to neural stimulation, accurate stimulation with high spatial and temporal precision are required for some neural prostheses such as retinal implant and cochlear implant. Unraveling the function and connectivity of neural circuits is essential for determining the specific target inside the brain area and assessing brain stimulation which involves deliberately modulating specific neurons accompanied by recording the responses. Reading out the neural activities are employed in wide area such as predicting cognitive intention, diagnosing diseases (e.g., EEG measurement used to diagnosis the epilepsy [6]) and guiding the therapy (e.g., a neural response telemetry for guiding stimulation parameters in the cochlear implant [7]). The gold standard method of the neural interface is based on the electrical method whose electrodes are placed in the vicinity of the target neural region [4,5]. Despite the robustness of the electrical neural interface, it requires invasive surgery when placing the electrode near the target neural region. Another drawback of electrical methods arises from the implanted electrode, whose position, shape and number of electrodes cannot be easily reconfigured or scaled after implantation. On top of that, though it depends on the distance between the electrode to the target neuron, poor spatial resolution due to electric charge spreading through the leaky tissue environment is a critical factor limiting the effective neural interface [8,9,10].

Due to the inherent technical challenges of electrical neural interfaces, there has been increasing demand of seeking new methodologies using alternative energy modalities such as optical [11], mechanical [12], magnetic energy [13,14]. Among them, neural interfaces using light have gained attention due to the superior spatial resolution whose optical energy can be focused down to the diffraction limit [15], which is in the range of micrometer. Moreover, since the photon could travel even through the air, an optical methodology is ideal for non-invasive neural interfaces.

**Figure 1:**
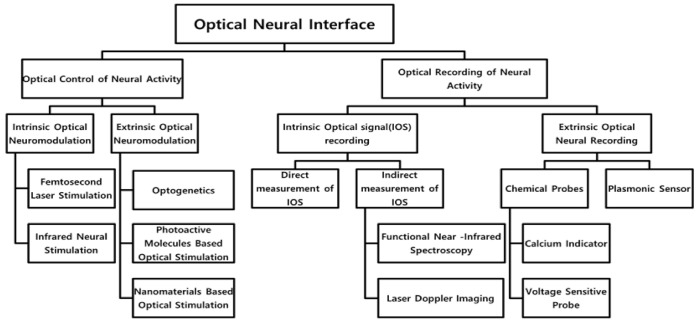
Optical neural interfaces consisting of an optical neural recording and an optical neural stimulation.

Optical neural interfaces include an optical control and an optical readout of neural activities, each of which can be subcategorized into an intrinsic and an extrinsic method depending on whether exogenous materials are applied or not. Since 1971 when Fork showed direct activation of abdominal ganglion neurons in Aplysia californica by shining a 488-nm laser, researchers have investigated the direct control of neural cells via optical illumination [16]. We classified this type of optical neural stimulation as an intrinsic neural stimulation as it requires no neural engineering prior to light irradiation [8]. In early 2000, researchers engineered the neuron by inserting exogenous materials (i.e., genes that eventually expressed to acquire light-sensitivity, nanomaterials, chemical compounds) to endow the light sensitivity of the neuron itself [11]. We entitled this type of neuromodulation as an extrinsic neural stimulation. In terms of the optical readout of neural activities, a direct optical recording has been investigated by measuring the spectral transmittance change of light [17]. Light itself also could record the hemodynamics allowing us to speculate the activities of the neural tissue [18]. Both types of recording can be grouped as intrinsic neural recording as it requires no labeling. Analogous to the optical neural stimulation, exogenous materials (i.e., fluorescent expressing genes, nanomaterials, chemical compounds) are also adopted to the optical neural recording to enhance the sensitivity, which can be named as an extrinsic neural recording [19]. In this review, we offer an overview of the current state of the art with regards to intrinsic and extrinsic optical neural interfaces (Figure 1). Moreover, the perspective of optical neural interfaces, especially in the view of clinical applications, are discussed.

## 2. Optical Control of Neural Activity

### 2.1. Intrinsic Optical Neuromodulation

Most neurons, for some exceptions, such as photoreceptor cells, typically do not respond to ambient light. However, illuminating the light at certain conditions can trigger neural activation. In 1971, Fork observed the neural spike upon shining a 488 nm laser at 160 W/mm^2^ onto the nerve cells in Aplysia [16]. Balaban et al. found that illuminating a 632.8 nm laser could depolarize the membrane potential as well as trigger the action potential in the subesophageal ganglia of Helix pomatia with a threshold intensity of 0.1 W/cm^2^ [20]. A bundle of central nervous fibers was excited when irradiating short pulsed (40 ns) UV light with the threshold intensity of 0.9 J/cm^2^ close to the photoablation threshold of 1 J/cm^2^ [21]. searchers have found the possibility that neurons themselves could become light-sensitive and intrinsically being stimulated by a certain condition of light.

#### 2.1.1. Femtosecond Laser Stimulation

One of the attempts to stimulate neurons optically with no exogenous materials is to use a femtosecond laser. The femtosecond laser emits a light pulse with a duration ranging from a few femtoseconds to hundreds of femtoseconds. Illumination of a femtosecond duration pulse, a nonlinear absorption of multiple photons occurs. This nonlinear photon absorption, called multiphoton excitation, exists where an optical power intensifies, especially only at the focused spot. In general, simultaneous absorption of two or three photons whose total energy is equal to the transition energy causes multiphoton excitation only in the target neuron, triggering nerve activation. Different mechanisms have been suggested depending on the stimulation intensity and durations. When neurons are irradiated with low light intensities with long durations of a femtosecond laser, the activation of neurons is triggered by the antioxidant Trolox. Upon stimulation, hydroxyl radicals, one of the antioxidants, are produced to depolarize the neuron, but at the same time these radicals could be related to inactivation or reversible damage of potassium channels. On the other hand, high intensity and short duration laser stimulation creates microholes in the plasma membrane and/or directly modulates the electric field via optical rectification that activates neural activity [22].

In 2001, Smith et al. monitored the calcium transient at the stimulation site when exposing a living HeLa cell using a femtosecond laser whose pulse duration, wavelength and average power are 140 fs, 780 nm and 30 mW, respectively [23]. Similarly, Hajime et al. used the femtosecond laser (wavelength: 750~850 nm, pulse duration: 130 fs, average power: 100~400 mW) to activate a pyramidal neuron [22]. A fast and reversible depolarization was monitored upon stimulation of neurons with high intensity and short pulse duration, whereas the continuous laser failed to induce membrane depolarization. In 2009, Liu et al. attempted the photostimulation of astrocytes, the major electrically non-excitable cells in the central nervous system, with femtosecond laser pulses providing the wavelength, the pulse duration and the average power of 800 nm, ~90 fs, ~60 mW, respectively [24]. The transient increase of intracellular calcium level was detected in both stimulated and the neighboring cells within 1 s after laser irradiation and confirmed its reproducibility. In their following study, they observed that stimulated and some neighboring cells generate calcium responses after irradiation and mapped the functional topological image of the neural circuit based on the calcium responses of postsynaptic neurons [25]. Later, the study of the astrocyte-to-neuron signaling in the hippocampal neural network in response to photostimulation with the femtosecond laser (wavelength: 800 nm, pulse duration: ~90 fs, power: ~30 mW) was demonstrated by Liu et al. [26]. In their experiment, femtosecond laser stimulation targeted to astrocytes reliably triggered intracellular calcium transients followed by the release of extracellular messenger which finally leads to neural activation. This astrocyte-to-neuron signaling was reliably observed when stimulating the cell with a laser power greater than 18 mW. Moreover, severe irreversible damage was detected for the laser power exceeding 40 mW. In 2012, Hosokawa et al. stimulated hippocampal neurons in the cultured neuronal network by a focused femtosecond laser (wavelength: 800 nm, pulse duration: ~100 fs, power: 30 mW) and measured single-cell action potential (AP) using a multi-electrode array (MEA) [27]. One-photon (300~600 nm continuous visible LED light) and two-photon (780 nm and 140 fs) stimulations were conducted by Jang et al. in a microfluidic culture system. They monitored that one-photon activation via caged-glutamate successfully elicited periodic spiking having a response time of 200 ms. In contrast, femtosecond stimulation provoked repetitive firing having a faster response time of 50 ms [28].

The stimulation using multiphoton has a significant advantage when it comes to high spatiotemporal resolution [26]. Due to the nonlinear dependency on the concentration of photons, the femtosecond laser triggers neural activities only at a focal point and could acquire high spatial selectivity [29,30]. The femtosecond laser stimulation, however, suffers possible cell death due to reactive oxygen species (ROS) produced during the stimulation and the need for the expensive and bulky laser source which both hinder its application to clinics [31].

#### 2.1.2. Infrared Neural Stimulation

Another type of intrinsic neural stimulation is an infrared neural stimulation (INS) where it aims to modulate neuronal activities by shining a pulsed infrared light. In 2005, researchers at Vanderbilt University first demonstrated that pulsed infrared light could evoke a compound nerve and muscle potential (CNAP and CMAP) in the rat sciatic nerve in vivo [32,33]. Infrared light ranging from 2.12 µm to 6.1 µm was employed and concluded that nerve bundles were safely stimulated with an average stimulation threshold of 0.32 J/cm^2^ which is only 16% of the ablation threshold. Following this finding, infrared neural stimulation (INS) was studied to optimize the stimulation parameters. Specifically, J. Wells et al. validates the effectiveness of INS by comparing the CNAPs and CMAPs of standard electrical stimulation methods [34]. They found no stimulation artifact in the measured CNAP after the optical stimulation with a duration of 250 µs, while strong electrical artifact was observed upon 5 µs long electrical stimulation. Superior spatial selectivity was also achieved during INS and a functional map of nerve fascicles to various muscles was identified. Laser radiant exposure and the peak CMAP showed linear-response relation analogous to the electrical response, which further substantiated the validity of INS [34]. McCaughey et al. determined the efficacy of INS in terms of wavelength using the rat sciatic nerve [35]. Laser diodes with a wavelength of 1540 nm, 1495 nm, 1540 nm and 2100 nm were used, and found that the 1495 nm source was the most reliable. However, meaningful comparisons over the wavelengths are difficult because of the large variation in the fiber diameter, the beam divergence and the pulse duration [36]. Peterson et al. showed high selectivity for INS, with 81% of scanned nerve being strongly spatially localized in the rabbit sciatic nerve using 1.3 J/cm^2^ at 1875 nm [36,37].

After the initial findings and validation of INS conducted primarily on the in vivo rat sciatic nerve, the application of INS in the cochlea as a tool for cochlear implant have been investigated. Izzo et al. implanted an optical fiber (core diameter: 100 µm) in 500 µm away from the target modiolus cochlea and shined infrared laser light having the pulse intensity, the wavelength, the duration and the repetition rate of 0.018 ± 0.003 J/cm^2^, 2120 nm, 250 µs and 2 Hz, respectively [38]. Light evoked compound nerve action potentials (CNAPs) were monitored with no evidence of neural damage. In the subsequent experiment, auditory neurons were optically stimulated with different laser diodes (wavelength: 1844~1940 nm, pulse duration: 5 µs~1 ms, repetition rate: 2~1000 Hz) [39,40]. The pulse duration was varied from 5 µs to 35 µs, and all of them successfully triggered the neural activation. Among those wavelengths tested, pulse duration shorter than 30 µs spatially confines the heat, which might cause tissue damage. In addition, Izzo et al. found the dependency of wavelength on tissue penetration in that the wavelength of 1.85 µm and 1.94 µm have ~1000 µm and ~85 µm penetration depth, respectively. Richter et al. investigated the optical threshold measured as energy per unit area to trigger CNAP in surviving spiral ganglion cells after inducing acute and chronic deafness induced by intracochlear application of neomycin and found that threshold remained unchanged for the pulse shorter than 200 µs while it increased for the pulse greater than 200 µs in the chronic model [41]. Izzo et al. expressed transcription factor, c-FOS, in activated neurons to identify the spatial area of the stimulated cochlea [42]. Immunohistochemical staining of c-FOS in the cochlea indicated that a small population of cochlear neurons was optically stimulated. In contrast, electrical stimulation led to an activation of larger population of neurons, confirming that the INS has better spatial selectivity than the electrical stimulation method. In 2014, Lee et al. stimulated cochlear neurons optically (wavelength: 1849 nm~1865 nm, duration: 5 µs~10 ms, repetition rate: 2~1000 Hz, and threshold: 169 mJ/cm^2^) after implantation of an optical fiber on the surface of the cochlear nucleus and validated whether the sound signal has been restored or not by recording the response from the central auditory system [43]. Optically evoked auditory brainstem responses (oABRs) occurred with 3 to 8 ms after stimulation. Reproducible oABRs were elicited when stimulating at thresholds of 169 mJ/cm^2^, with 50 µs pulse duration and 5 Hz repetition rate. In 2012, Schultz et al. showed that light exposure in the wavelength ranging from 400 nm to 2000 could elicit CNAPs in the cochlear neurons [44]. CNAPs were provoked with nanosecond pulses similar to acoustic stimulation, while no response to the laser stimulation, in any case, was observed after complete deafening of the cochlear. The first behavioral study was conducted by Matic et al. in 2013 after chronically implanting an optical fiber targeting the spiral ganglion neurons in the freely moving cat [45]. Cats responded and turned their head toward the direction of the optical stimuli, indicating that INS successfully restored the hearing. No optically evoked response was monitored once the auditory nerve of deafening animals was cut [46].

INS has been applied to other excitable tissue. Teudt et al. stimulated the gerbil facial nerve using Ho:YAG laser (wavelength: 2120 nm, core diameter: 600 µm, pulse duration: 250 us, and frequency: 2 Hz) [47]. Optically evoked responses were observed when shining a laser with various radiant exposures ranging from 0.71 J/cm^2^ to 1.77 J/cm^2^ and found that their responses resembled that observed when using electrical stimulation. The heart is another target of INS. In 2010, Jenkins et al. demonstrated that the heart of an embryonic quail could be paced by illuminating the 1975 nm laser light (0.88~4.33 J/cm^2^) with no damage in tissue [48]. Finally, the brain is being explored as a potential target of INS. Cayce et al. presented in vitro study in rat thalamocortical brain slices using free-electron laser [49]. Increasing the wavelength from 2.41 µm to 5.3 µm as well as the repetition rate from 7.5 Hz to 30 Hz resulted in low threshold radiant exposure measured as energy per unit area. As in vivo study, Cayce et al. optically stimulated a somatosensory cortex of rats using the laser whose wavelength, repetition rate and radiant exposure are 1875 nm, 50~200 Hz and 0.01~0.55 J/cm^2^, respectively.

Since INS requires just an optical element equipped with an inexpensive laser source with no exogenous material to be injected, it is a low-cost and simple technique. Moreover, it does not generate any ROS that might damage the tissue. However, INS suffers from the safety issue caused by the accumulated heat due to the high water absorption. Due to its safety issue caused by bulk tissue heating, many studies were made to determine the optimal stimulation level to guarantee safety [50]. Therefore, despite its intrinsic properties and relatively simple technique, innovative breakthrough to avoid bulk tissue heating is essential to bring INS into clinics.

### 2.2. Extrinsic Optical Neuromodulation

Since most neural tissues do not respond to ambient light and the light detecting properties are not their primary function, the required light exposure for neural activation is relatively high and might cause tissue damage. Therefore, alternative techniques to lower the stimulation threshold have been developed by adding exogenous materials to the neural tissue.

#### 2.2.1. Optogenetics

Optogenetics is a technique that genetically introduces light-sensitive ion channels to neurons and thereby allows them to be switched on or off optically with high spatial and temporal resolution [51]. The term “optogenetics” contains two complementary approaches: monitoring neuronal activity using genetically encoded fluorescent reporters (sensors) and controlling neuronal activity using genetically addressable light-activated tools (actuator) [52]. Here, optogenetic as a neural controller is reviewed by dividing it into two groups depending on the number of components being involved: a multicomponent system and a single-component system.

The first genetic approach was to employ a phototransduction process incorporating multicomponent systems in animal vision. Khorana et al. observed light-dependent ionic currents in Xenopus oocytes transfected with bovine rhodopsin, membrane-embedded light-sensitive photopigments [53]. Each rhodopsin molecule consists of an opsin protein covalently bound to a chromophore. Upon illumination, the bound retinal molecule is isomerized, which induces conformational changes in the opsin backbone. Subsequently, a G-protein signaling pathway is triggered, followed by non-specific cation channel activation [52]. In 2002, Zemelma et al. co-expressed photoreceptor genes (rhodopsin + arrestin + Gqa, a combination known as “charge”) in cultured mammalian hippocampal neurons and monitored the light-evoked neural firing but suffers intrinsic drawbacks such as slow and variable activation and deactivation kinetics [54]. To overcome the inherent limitation of the slow and temporally imprecise metabotropic nature of opsin signaling, Deisseroth et al. engineered chimeric receptors by replacing the intracellular loops of the bovine rhodopsin with the specific adrenergic receptors [52]. They could optically activate the intracellular pathways to condition the preference of region in freely moving mice. Mice stayed longer in certain locations, where they received the monoaminergic input as a reward by optical stimulation [55]. Stefan’s group produced a light-activated receptor recruiting the signaling cascade of a specific serotonin receptor. They observed 8~9 mV membrane hyperpolarization responses to a 1 s pulse of 486 nm wavelength of light after expressing the light-activated G-protein-coupled receptor [56].

The study of light-driven ion transport in the archaea could become an ideal light-gate actuator involving a single component rather than an effector protein that is activated by a multicomponent signal cascade system [52]. Channelrhodopsins of the common rhodopsins having intrinsic light-gated ion conductance change was obtained from the eyespot in the green algae Chlamydomonas [57]. Nagel group cloned two channelrhodopsins in 2002~2003: the first one is the Channelrhodopsin-1 (ChR1) that is selectively permeable to protons (H^+^), and the other one, a Channelrhodopsin-2 (ChR2), is permeable to other cations, which both generate depolarizing current upon optical illumination [58,59]. These channel-like proteins were introduced to the Xenopus oocytes and demonstrated that rapid photocurrents responses are generated within tens of microseconds upon illumination with blue light (wavelength: 450~500 nm) [57,58]. The feasibility of the single-component optogenetic approach was demonstrated by Boyden et al. in 2005 [60]. After the expression of ChR2 in cultured hippocampal neurons, neurons were illuminated by blue light (wavelength: 490~510 nm and optical intensity: 8~12 mW/mm^2^), leading to ChR2-mediated photocurrents with millisecond precision. A new channelrhodopsin, cloned from two species of the green algae, has been successfully used in mammalian neurons: VChR1 from a volvox carteri [61] and MChR1 from mesostigma viride [62].

Rhodopsins are also used to inhibit neural activities. Other types of microbial rhodopsins, such as a bacteriorhodopsin, a photorhodopsin and an archaerhodopsin were discovered recently in bacteria [63] and some eukaryotes [64]. Similar to the channelrhodopsins, these microbial rhodopsins are also single-component, light-sensitive proteins but working as ion pumps. Upon light illumination, these proteins extract protons from the cytoplasm to extracellular space resulting in hyperpolarization of membrane potential. Halorhodopsin, on the other hand, transports chloride ions to maintain the osmotic balance of halobacteria. When shining light, chloride ions are pumped into the cell leading to membrane hyperpolarization [65]. Both types generate a hyperpolarizing photocurrent which can be used to silence the neuronal activity. Since these inhibitory pumps operate at different wavelengths, these proteins can be coexpressed with ChR2 to achieve bidirectional control in the same cell. In 2007, Zhang et al. coexpressed ChR2 and the NpHR (halorhodopsin) from Natronomonas pharaonis in acute brain slices and Caenorhabditis elegans [66]. Irradiation of acute cortical sections using 473 nm blue light (50 pulses for 15 ms at the repetition rate of 10 Hz) for ChR2 activation but without 593 nm yellow light (continuous illumination for 6 s) NpHR activation results in the intracellular calcium transient increase. C. elegans, which expresses ChR2 and NpHR, show contraction and relaxation when exposed to blue and yellow light. However, a high level of expression impairs intracellular localization. After extensive tests of various light-gated inhibitory ion-pumps to improve their membrane localization, eHpHR3.0, Arch and ArchT emerged as promising light-activated silencers [67].

Optogenetics can reliably and robustly activate or inhibit particular cell types and specific neuron circuits with high temporal resolution. Despite cell type specific targeting with high spatiotemporal precision, optogenetics has limitations such as the inevitable gene therapy which is the biggest concern in terms of safety and ethical issues. To date, many clinical trials, especially building retinal prosthesis devices using optogenetics have been conducted, still inherent potential threat that optogenetic cannot be employed in humans remains [68].

#### 2.2.2. Photoactive Molecules Based Optical Stimulation

Another technique to optically activate the neurons is based on light activated chemical molecules. One type of these photoactive molecules are neurotransmitters that are held in photosensitive cages but can be liberated upon exposure to light [69]. Caged glutamate is one of the most commonly used molecules for neural stimulation. Another approach is to use the bistable molecules enabling conformational change upon light irradiation. Upon shining the light on the photoactive molecule with different wavelengths, molecular shape changes from trans to cis or vice versa [70].

With caged neurotransmitters, photoactive molecules-based optical stimulation has become popular in neuroscience [71]. Photoactive chemistry techniques can modulate neuronal activity by activating specific receptor proteins using synthetic photoconvertible ligands. The first experimental uncaging glutamate on dendrites was conducted by Kandler et al. on CA1 pyramidal neurons in slices [72]. They reported that photodegradation of caged glutamate blocking the synaptic transmission reduced the subsequent response to glutamate. Dalva and Katz used this technique to map the developing synaptic connections in the primary visual cortex of ferrets [73,74]. Schiller et al. measured calcium level in dendritic spines in layer 5 cortical pyramidal neurons using a 361 nm UV-laser beam (shuttered pulse: 1 ms and optical power: 1~2.2 mW) induced photolysis of caged glutamate. Most of the calcium influx that reacts only with glutamate was through voltage-gated calcium channels [75]. They then reported that local dendritic spikes were triggered after uncaging with a 1 ms shuttered pulse whose wavelength is 341 nm [76]. Dendrite spikes were spatially confined to the dendrite activation region. In 2005, Lima and Miesenbock presented the first example of an optically remote-controlled animal to elicit specific behaviors in fruit flies [77]. Brief, 355 nm UV illumination (optical power intensity: 8 mW/mm^2^, pulse duration: 150~250 ms and repetition rate: 100 kHz) on flies elicited activity in small sets of motions such as leg extension, jumping, wing opening and high-frequency wing flapping. This technique was used to control nicotinic receptors [78], ionotropic glutamate receptors [79], potassium channels [80,81,82], chimeric potassium-selective glutamate receptors called Highlighter [83].

Fortin et al. proposed a photosensitive protein called Photoswitch Affinity Label (PAL) as a bistable molecule that targets voltage-gated K^+^ channels to block the channel [70]. Covalent binding of PAL to a channel is facilitated by ligand binding. After the photoswitch is covalently bonded, QA (a quaternary ammonium group in PAL) reaches the pores only when the azobenzene (part of PAL) is in the elongated trans form and blocks the ion conduction. Meanwhile, channels can be unblocked by exposing azobenzene to 360~400 nm light, which photoisomerizes trans to cis. Exposure of long-wavelength light (wavelength: 450~560 nm) reversibly converts cis to trans. They showed that PAL treatment confers light sensitivity on K^+^ channels in isolated rat neurons and in intact neural structures from rat and leech after illumination with 0.4 mW/mm^2^ and 3.5 mW/mm^2^ for 380 nm and 500 nm of light, respectively. Large extrinsic K^+^ currents were measured in PAL-treated, cultured hippocampal neuron after exposing 500 nm wavelength of light. Action potential was immediately suppressed upon shining 380 nm light while high-frequency firings resumed with continuous illumination with 500 nm light in their experiment.

Photoactive molecule-based photostimulation has advantages without the need for genetic engineering or exogenous gene expression. It can focus on intracellular structure, a single cell or diffusely project to regulate the activity of many cells at the same time. However, several photoactive molecules require blue shifted or UV light for activation, causing potential tissue damage when implanted chronically [84]. One major drawback of the photochemical technique is the necessity of either delivering the ligand or conjugating the photo tethered ligand to the target protein. Since these cages have potential toxicity, their potential use in clinics is restricted. Even worse, difficulties in binding these molecules in the living sample also hinder applications in vitro, ex vivo and tiny organisms (e.g., tissue culture, brain slice, fruit flies, zebrafish larvae).

#### 2.2.3. Nanomaterials Based Optical Stimulation

Even though genetic modification could modulate neural activation with low radiant exposure power, it still suffers ethical and safety issues regarding genetic modification. Adding photoactive molecules, however, alleviates this concern but the phototoxicity of the chemical compound and the use of UV-light are remaining issues. Therefore, nanomaterials are introduced to circumvent these limitations by modulating neural activities in two ways: photothermal and photovoltaic optical neural stimulation.

Compared to the INS method suffering possible tissue damage due to high water absorption of infrared light, the use of NIR light lying in the range of the water window alleviates the possible concern of thermal damage [85]. Instead, gold nanomaterials are placed near the target neural tissue as efficient light absorbers to create localized thermal heat avoiding bulk tissue heating. When the light frequency is tuned to the surface plasmon resonance frequency of gold nanoparticles, they efficiently absorb the light to create localized thermal heat [86]. The local temperature elevation at the membrane produces a brief capacitive current and/or sustained current through a temperature-sensitive ion channel which both elevates the membrane potentials for neural activation [87].

In 2013, C. Paviolo et al. first showed that the 780 nm laser exposure of gold nanorods could induce intracellular calcium transient of neuroblastoma x rat glioma hybrid cells. Strong intracellular calcium transients were obtained upon 0.33 J/cm^2^ exposure of light for 50 ms [88]. In their following study, neuroblastoma cells incubated with gold nanorods showed 20% increased neurite growth upon irradiation of 780 nm laser (1.25~7.5 W/cm^2^) for 1 min compared to that without the gold nanorods incubation [89]. The potential that the nanomaterials, especially the gold nanoparticles incorporated with near-infrared (NIR) light, could modulate the cellular activation and lead to neurite outgrowth initiated the introduction of gold nanoparticles to neurons. In 2014, J. Yong et al. showed the 780 nm laser (peak optical power: 90 mW, pulse duration: 25 ms) induced action potentials of the primary cultured auditory neurons incubated with gold nanorods in vitro [90]. No stimulatory response was observed at the control neuron without nanoparticles and with gold nanospheres whose surface plasmon resonance peaks were detuned (located at ~525 nm), inferring that the photothermal effect is the main source of this neural activation. Similarly, K. Eom et al. demonstrated that 980 nm laser illumination onto the gold nanorods treated rat sciatic nerve in vivo elicited CNAP [9]. Laser radiant exposure and the CNAP showed a linear relationship with 5.7 times higher responsivity and lower stimulation (0.159 J/cm^2^) threshold than obtained without gold nanorods. Gold nanoparticles are decorated with cell-targeting ligands (antibodies) for strong conjugation [91,92,93,94]. Carvalho-de-Souza et al. showed in vitro cultured hippocampal neuron and ex vivo brain slice that neurons can be stimulated by 532 nm light (laser intensity: 31 kW/cm^2^, pulse duration: 1 ms) with gold nanospheres coated with Ts1, a neurotoxin specifically targeting voltage-gated sodium channel [91]. Eom et al. showed that the stimulation of the motor cortex after injection of gold nanorods elicited the whisker movement [92]. In addition to the antibody enabling specific antigen targeting, non-specific binding of gold nanoparticles to the cell membrane using cholesterol [94], high-density lipoprotein [93], amine-terminated polyethyleneglygol (NH2-PEG) [95] induced neural activation. Not only the neurons but also the glial cells [96] are also responsive to the optical stimulation accompanied by gold nanomaterials. Increased stimulus duration at the reduced intensity affects the physiological response in a different way, inhibiting the natural spontaneous response [95,97,98,99]. Yoo et al. found that inhibition of neural activities occurred less than 1 s of NIR illumination (15 mW/mm^2^), and even 30 min of illumination could reversibly suppress the neural activities [95]. They confirmed that the TREK-1 ion channel known as the temperature-sensitive potassium channel is responsible for the neural inhibition upon membrane-localized photothermal effects.

Photovoltaic neural stimulation is another type of nanomaterial based extrinsic optical neural stimulation strategy which stimulates neurons by generating electric voltage in response to light. This method has widely been employed in building ultrathin retinal implants for restoring the vision of the blind [100]. Two types of photovoltaic neural stimulation have been developed depending on whether an external power supply is required or not [100,101,102]. In this review, we will focus on the photovoltaic approach that does not require an external energy supply for stimulation. In 2012, K. Mathieson et al. developed a multi-channel micro-photodiode array capable of generating charge-balanced electrical stimulation pulse [102]. Upon illumination of 905 nm light for 1 ms with an intensity of ~0.5 µW/mm^2^, light-driven spikes were elicited from extracted retinal ganglion cells. Y. Mandel et al. further validated the functionality of the photovoltaic stimulation as a possible candidate for the retinal prosthesis and confirmed that NIR protection on the photodiode array implanted subretinally evoked cortical responses analogous to those acquired after visible light stimulation in normal rats [103]. In addition to the photodiode, various other nanomaterials are developed and employed. J. Tang et al. showed that subretinally implanted gold nanoparticles coated with titania (Au-TiO2) nanowire arrays could stimulate retinal neurons upon visible light stimulation with a duration of 1 s and the threshold intensity of ~0.5 µW/mm^2^ [104]. Moreover, the activities of cardiac cells were controlled by photovoltaic stimulation. Cardiomyocytes that were grown on the reduced graphene oxide(rGO)-coated coverslips showed increased electrical activity upon light illumination (green, 2.1 mW/mm^2^) [105].

The use of NIR light to avoid the bulk tissue heating caused by strong water absorption greatly reduced the potential tissue damage due to heat which was the major concern of the INS. Targeting the exogenous materials to the neuronal membrane known to be responsible for the thermal neural activation and shining the NIR light onto it creates spatially confined heat near the membrane. In the photovoltaic device, it is a robust and reliable technique because its neural stimulation mechanism is based on electrical stimulation. However, the necessity of implantation of exogenous material and potential tissue damage due to thermal heat restricts its use in animal studies.

## 3. Optical Recording of Neural Activity

Various neural recording techniques have been developed. Up to date, electrophysiology has been the prevalent method due to its wide range of neural applications, capturing neural spikes from individual cells to compound network responses from small neuronal populations. Depending on the spatial separation between neurons and electrodes, the types of neural response recorded varies. Intracellular recording using patch clamp techniques measures the current of a single ion channel and precisely monitors the action potentials [106]. Extracellular recording, whose neurons to microelectrode separation is small, records the spikes from the individual neuron [107]. Macroscopically, electroencephalography (EEG) using the patch electrode attached to the scalp captures the oscillatory local field potentials (LFPs) from the subpopulation of neurons non-invasively [107]. Despite its variability, no technique enables the recording of neural activities non-invasively with high spatial resolution. Non-invasive brain imaging methods using magnetic fields such as magnetoencephalography (MEG) [14], function magnetic resonance imaging (fMRI) [108] and positron-emission tomography (PET) [109] have been employed. However, these techniques suffer low spatial resolution and complexity of the equipment. For these reasons, there have been increasing demands for the development of non-invasive neural recording methods capable of functional imaging with high spatial selectivity using simple imaging systems.

### 3.1. Intrinsic Optical Signal (IOS) Recording

#### 3.1.1. Direct Measurement of IOS

Light interacts with neural tissue, and their light properties change depending on the activation of neurons. This interesting phenomenon was first discovered in 1949 by Hill et al. where they stimulate nerves at the frequency of 50 Hz for 5 s whose individual stimulus is sufficient to generate action potentials [110]. They found that the opacity, especially the scattering, fluctuates upon nerve stimulation. Stepnoski et al. reexamined the coupling between light scattering and the membrane potential in the cultured neurons from Aplysia and monitored the changes in the membrane potential using a dark-field microscope [111]. Linear relation of light scattering with respect to the membrane potential was obtained and showed that the trace of light scattering followed that of membrane potential change. Similarly, Rector et al. showed that Schaffer collateral stimulation leads to light scattering changes with a similar time course of evoked potential in the hippocampal region CA1 [112]. The fast optical response was monitored by Lee et al. after they electrically stimulated the brain slice sample [113]. Since the intrinsic noise of the system is large (~10^−3^) compared to that of the signal (~10^−4^), the 500 optical transmittance data were averaged to increase the SNR. They found the spectral dependence of this transient optical response whose relative changes were higher at 1250 nm than 830 nm. Even though the theoretical origins underlying optical properties change during neural activation, many researchers elucidated that light scattering changes possibly originated from the volumetric as well as the refractive index change during neural activation [17,113].

One of the parameters determining the scattering change in the tissue is the average time traveled by the photon from the source to the detector [17]. Increased scattering in the tissue results in random motion, and thereby the distance traveled in the tissue has been increased before reaching the detector. Time delay, usually in the order of picoseconds, is calculated by cross-correlating the detected signal and reference light modulated at the high frequency [113,114,115]. Gratton et al., employed a near-infrared light (715 nm) LED source incorporated with detector fiber positioned above the central portion of the visual field (area 17) of the human subjects. Upon visual stimulation, the increased time delay of photons was monitored, having a peak at 100 ms after stimulation [114]. In the following study, Gratton et al. compared the visually evoked event-related optical signal (EROS) with the gold-standard fMRI response measuring hemodynamics and VEP signals [113]. The EROS signals were colocalized (subcentimeter) with the fMRI response but with shorter latency, while EROS temporally coincided (subseconds) with the signal recorded with the VEP.

Since the typical latency of direct measurement of IOS does not exceed hundred milliseconds, indirect IOS is regarded as a ‘fast optical signal’ and postulated as a fingerprint of the membrane potential changes. Moreover, due to its complete non-invasive properties, this method can be an alternative tool for the EEG, which is widely used in the clinical study including diagnosis, prognosis and therapy of many neurological disorders [116]. Even though its superior temporal resolution, robustness over other optical neural recording techniques is under debate [117,118]. Steinbrink et al. found that EROS were reliably recorded from only a few human subjects even though they used optical instruments that could detect the small intensity changes (2 × 10^−3^%) [117]. Moreover, in 2009, Radhakrishnan et al. detected no EROS after visual stimulation in the live monkey even after averaging thousands of EROS in the same animal, whereas hemodynamic as well as electrical evoked potential was always recorded [118]. Unless the noise level of the optical instrument is significantly improved, it seems that direct measurement of IOS could not replace the conventional electrophysiological methods that are already used in the clinics.

#### 3.1.2. Indirect Measurement of IOS

While direct measurement captures the optical properties variations associated with neural activation, indirect measurement of IOS investigates the concentration changes of marker substances linked to brain activities. Since oxygen metabolism is the primary source of brain activities, the measure of the oxygen metabolism level as well as other related to it indirectly represents the brain activities. For instance, a local increase in cerebral hemodynamics is triggered as active neurons demand oxygen carried by oxyhemoglobin (HbO2) [119]. As neuronal activities and hemodynamics are tightly coupled, it is termed neurovascular coupling. Moreover, the concentration of HbO2 increases while the concentration of deoxyhemoglobin (HbR) decreases because of the intensified supply of fresh blood containing abundant HbO2 to the activated brain region [120]. The hemoglobin related hemodynamic response is called the “blood oxygenation level dependent” (BOLD) response [18].

##### Functional Near-Infrared Spectroscopy

Based on the fact that the NIR light transparency of biological tissue, Frans Jöbsis demonstrated the in vivo monitoring of HbO2 and HbR in the brain for the first time [121]. Near-infrared spectroscopy (NIRS) developed by Jöbsis led to the possibility of monitoring the changes of brain oxygenation. In 1985, NIRS was first brought to clinics for monitoring cerebral oxygenation on newborn cerebrovascular patients [122]. First functional NIRS (fNIRS) studies performed on adult humans were reported independently by Hoshi et al. [123], Kato et al. [124] and Villringer et al. [125]. The term “functional” in fNIRS means that the NIRS is used to monitor neural function. Hoshi et al. monitored the oxygenation change in the frontal region of the brain as an indicator of HbO2 and HbR changes during mental work [123]. Using NIRS instruments consisting of three laser diodes (780, 805 and 830 nm), increased HbO2 while decreased HbR were monitored when the subject was solving difficult arithmetic. Kato et al. measured the visual cortical function using commercial NIRS instruments (NIR-1000, Hamamatsu) [124]. Photic stimulation of human subjects elicited elevation of both total hemoglobin (tHb) concentration reflecting total blood volume and HbO2 in the occipital region, while no changes were monitored in the prefrontal cortex. Similarly, Villringer et al. found that cognitive task and visual stimulation trigger oxygenation changes (increase in HbO2 and tHb while decrease in HbR) in the frontal cortex and occipital cortex [125]. Peak hemodynamic response was monitored 10 s after visual stimulation showing that neurovascular coupling based indirect monitoring of IOS is ~100 times slower than direct measurement of IOS mentioned in Section 3.1.1. These preliminary observations confirmed that NIRS could be used to detect human brain activity non-invasively.

The simple principle of probing HbO2 and HbR underlies the fact that light absorption of HbO2 and HbR differs depending on the wavelength. NIR light with a wavelength greater than 800 nm is mainly absorbed by HbO2, while HbR greatly absorbs the NIRS light below 800 nm [126]. Upon shining multiple wavelengths of light onto the brain, light attenuates in the brain and the degree of attenuations are measured to find the concentration changes of HbO2 and HbR using Beer-Lamberts’ law [18,126]. Based on the aforementioned method, the continuous-wave (CW) fNIRS measures the attenuation of incident light after shining light with constant intensity. Meanwhile, the frequency-domain (FD) and the time-domain (TD) methods obtain the absolute values of HbO2 and HbR by illuminating amplitude modulated light and short light pulse onto the brain tissue, respectively [18]. Since FD and TD fNIRS measure phase delay and broadening, respectively, as well as the attenuation of the incident light, both methods detect the absolute values of HbO2 and HbR.

Due to its portability, low noise level and robustness against motion artifacts compared to other non-invasive neural recording techniques such as fMRI, EEG and PET, fNIRS is well-suited in monitoring brain activities under physical activities [126]. Subjects with metallic implants are able to use fNIRS to monitor their brain activity while fMRI could not. However, fNIRS suffers low penetration depth, limited to cortical layer, as the maximum depth of brain region to be monitored is limited, in general, less than half of the source to detector separation [127]. Moreover, the hemodynamic change originating from extra-cerebral tissue such as the scalp, skull and frontal sinus may contaminate the hemodynamic signal from the brain tissue [128]. fNIRS also suffers from low spatial resolution (~1 cm) compared to fMRI (~4 mm), known as the gold standard method of non-invasive functional brain imaging. Even though its limitation, fNIRS has wide potential clinical applications such as language mapping [129], basic functional neuroimaging research [130], epilepsy [131,132,133], Parkinson’s disease [134], pain assessment [135] and autonomic dysfunction [136].

##### Laser Doppler Imaging

Laser doppler flowmetry is a non-invasive tissue blood flow measuring technique using a doppler shift of light. Upon shining light to a moving particle, the light is being scattered from it and is shifted in frequency depending on the velocity [137]. As the particle moves towards the detector, the blue-shifted light is monitored at the detector, whereas a red-shifted signal is observed for the particle receding away from the detector.

The application of laser doppler flowmetry (LDF) on monitoring blood flow dates back to 1972 when Riva et al. first demonstrated the velocity distribution of whole blood flowing in a 200 µm diameter capillary using the LDF [138]. Since then, the LDF has been used to characterize cerebral hemodynamics for the functional brain imaging [139,140,141]. Since LDF used in previous publications employed a single probe, all the results were limited to the single point observation. 2-D spatial map of hemodynamics was achieved by incorporating the scanner into the single point LDF. The scanner scans the optical beam along with the brain and measures the doppler shift in 2-dimension [142]. The laser doppler imaging (LDI) technique using a scanner typically requires minutes while the subject is immobilized. In the early 2000 s researchers employed a fast CMOS camera as a detector in the LDI to acquire full-field doppler perfusion without a scanner [143,144,145]. LDI instruments were developed to acquire real-time blood perfusion by incorporating high-speed cameras with the LDI systems. Rabbe et al. integrated CMOS-based LDI into the surgical microscope for functional brain imaging during neurosurgery [146]. During the awake surgery, patients were asked to perform specific tasks and a 2-D map of blood perfusion was acquired. Blood perfusion was increased by 10~20% with respect to the baseline and its region of activation well correlated with that found by preoperative fMRI imaging and intraoperative electrocortical stimulation. Laser doppler methods have widely been applied to monitor peripheral blood flow for diagnosis of various diseases (e.g., diabetes [147], rheumatic disease [148]), burn assessment, functional imaging of optic nerve through eye [149] and cerebral functional brain imaging for intraoperative neurosurgery [146]. In contrast with the fNIRS measuring the hemodynamics of the brain cortex usually on the scalp, typical LDF monitors the cortical hemodynamic of an exposed brain. This restricts LDF to a variety of clinical applications.

##### Laser Speckle Contrast Imaging

When a coherent light illuminates the diffusive medium, the scattered light produces a random granular shaped interference pattern called speckle [150,151,152,153]. When the medium is stationary, the speckle pattern does not change in time. However, if the medium is moving, this will cause the speckle pattern to fluctuate. Imaging the time varying speckle pattern using a camera with a certain exposure time would create a blurred speckle pattern due to the integration of time varying speckle signal over time [154,155,156,157,158]. By imaging the speckle pattern over time and quantifying the degree of blurring, a laser speckle contrast imaging (LSCI) technique obtains the 2-D map of medium flow [155,156,158].

The use of laser speckle to measure the flow was first demonstrated by Fercher et al. in 1981 [154]. By applying the optical spatial high pass filtering technique of a single exposure speckle photography, they were able to obtain a spatial map of retinal blood flow. After the development of digital photography, the LSCI technique became much simpler, avoiding the photographic process and was employed in various applications, especially in the biomedical field, such as investigating migraines [155], cerebral blood flow monitoring [151,156], wound and burn assessments [157,158] and retinal imaging [159]. Since the LSCI acquires a 2-D flow map of cerebral blood flow, it has been employed for functional mapping of brain activities. In 2005, Dunn et al. monitored the hemodynamic, including the blood oxygenation near the somatosensory cortex while stimulating forepaw and whisker [160]. Contrary to the previous publications which monitor the cerebral blood flow using a single wavelength of laser, multiple wavelengths of the laser are adopted to simultaneously monitor the cerebral blood flow and oxygen metabolism. Even though LSCI lacks absolute measurement of blood flow, scanning-free full-field imaging of hemodynamics brought LSCI to functional imaging of brain activities. Analogous to LDF, LSCI is widely used in rheumatology to determine the state of sclerosis [161], burn assessment, blood perfusion monitoring during intraoperative surgery [161], imaging ocular blood [162] and functional brain mapping [163]. However, the scalp and skull should be removed during the functional LSCI in the brain cortex [163], limiting wide clinical applications.

### 3.2. Extrinsic Optical Neural Recording

#### 3.2.1. Chemical Probes

##### Calcium Indicator

Calcium ions are engaged in neuronal activity in many aspects. Calcium influx at the presynaptic terminal triggers the exocytosis of the neurotransmitter. In the postsynaptic terminal, activity-dependent synaptic plasticity is induced by a calcium transient. Moreover, intracellular calcium regulates the gene transcription in the nucleus [19,164]. Depending on the process that the somatic calcium signal governs, the overall calcium signal lasts for milliseconds and hours when cells are activated [164,165]. Since intracellular calcium transient is strongly related to the action potential, the number and timing of action potentials and the synaptic input can be quantified by measuring the intracellular calcium transient [166]. Thus, recording intracellular calcium signals is essential for monitoring neural activity.

The first calcium indicator was aequorin, a calcium-binding bioluminescent protein isolated from a jelly-fish in 1962 [167], which was later used to detect the membrane potential of the muscle fiber [168]. Ashley et al. detected S shaped rising phase of calcium transient just after the onset of electrical stimulation followed by an exponential decay with a time constant of 80 ms after the cessation of the stimulus. However, due to the cumbersome delivery of calcium indicators to the intracellular region using fine capillary [169,170], it has not been widely used to detect somatic calcium signals. More sensitive fluorescent calcium indicators were developed by hybridizing calcium chelators such as the EGTA and the BAPTA that selectively bind to calcium ions over other metal ions [171]. Tsien et al. used a Quin2, a chelator based calcium indicator, to measure the cytoplasmic free calcium concentration of an intact mouse and pig lymphocytes [172]. Acetoxymethyl ester endows the Quin2 with a cell permeable property and thus trapping the impermeable Quin2 in the cytoplasm once it is incubated with the cell. After loading the dye, its fluorescent (excitation: 339 nm, emission: 492 nm) signal increases about sixfold over the full range of the intracellular calcium concentration (100 µm). Over the years, many more calcium indicators with calcium affinity and a wide range of fluorescent wavelengths were developed such as fura-2, indo-1, fluo-3, fluo-4 and Oregon Green BAPTA.

Despite the success of chemical calcium indicators, it suffers from uneven and nonselective distribution of dyes inside the tissue as well as inevitable invasive loading procedures [173]. Genetically encoded calcium indicators (GECIs) were developed to solve these problems. Aequorin calcium probes are targeted to specific subcellular locations by using its cDNAs with a targeting sequence [174]. A GCaMP is another type of GECI based on a single green fluorescent protein (GFP) with calmodulin (CaM), a calcium responsive element [175]. Even though GCaMPs are widely used to probe cytosolic calcium level, measuring calcium level in other subcellular organelles is problematic because its fluorescent intensity is affected by the acidic environment [173]. For this case, a “chameleon” type indicator has been developed for subcellular monitoring of calcium signals. The GECI is powerful in that it allows specific expression by using specific virus serotypes or promoters or the Cre recombinase driver with transgenic animals [176,177].

All the calcium probes mentioned above share common mechanisms in that conformational changes are accompanied by the calcium binding to the indicator, making dye fluorescent upon illuminating with a specific wavelength of light [164]. Even though imaging intracellular calcium signals using dye offers 2D maps of neural activities with high SNR requiring no averaging process, there are several limitations in that the fast membrane potential changes cannot be monitored, and subthreshold signals cannot be detected. In addition, calcium signals are highly dependent on the intrinsic and extrinsic calcium buffer [178]. Since genetic modification or the injection of exogenous compounds into the target tissue are necessary, they are currently limited to the research field using animal models.

##### Voltage-Sensitive Probe

One of the main physiological changes accompanied by the neural activation is the membrane potential change resulting from ion movement across the membrane. Since monitoring the membrane potential is the most direct and exact method to determine the neural function, neuroscientists have traditionally relied on electrophysiology using electrodes. In this sense, optical neural imaging of membrane potential has been investigated. The first successful fluorescent imaging of membrane potential was performed using organic voltage-sensitive dye (VSD) in invertebrate samples where the fluorescent signal changes upon nerve activation [179,180]. Subsequently, VSD was used to image the activity of the mammalian cultured-neuroblastoma [181] and brain slice [182]. Application of VSDs to image population of neural circuits in vivo initially suffered from several problems such as a low signal due to the inefficient dye and the considerable noise due to the respiratory and the heart beat [183]. Development of efficient dyes (e.g., RH-414, RH-704 and RH-795) as well as noise compensation techniques enabled in vivo imaging of neural activities in the rat somatosensory cortex [184] and the monkey visual cortex using the VSD [185].

To optically image the membrane potential using VSDs, first, the sample is stained with VSDs attached to the plasma membrane. VSDs undergo optical properties change, e.g., emission and excitation spectrum changes, as a result of the membrane potential variation. In principle, the VSD relies on several different mechanisms such as redistribution, reorientation and electrochromism [178]. The most widely employed VSDs operate based on the electrochromism, where they change their electronic structure depending on the external electric field exerted by the membrane potential [186]. Electronic structure change alters the fluorescent spectrum, leading to fluorescent intensity variation as the membrane potential changes. Since the intramolecular charge redistribution is only involved, the electrochromism is fast to record the action potential. Moreover, combining existing VSDs with fluorescence resonance energy transfer (FRET) enhances the fluorescence signal [185].

Analogous to the calcium indicator, organic VSDs are not suitable for the selective binding of the target tissue [185]. To overcome this limitation, a genetically-encoded voltage indicator (GEVI) has been developed. The fluorescent protein is combined with the voltage sensor, which undergoes a conformational change upon membrane potential change that alters spectra of the fluorescent protein [187]. The first FRET voltage-sensitive fluorescent protein (VSFP) is a VSFP2.3 which is used to image the action potentials in cultured neurons, acute brain slices and the somatosensory cortex of living mice [188]. Other types of VSFP were developed: microbial rhodopsin-based GEVI and chemogenetic indicators to overcome the limitations of conventional GEVI such as slow kinetics, poor photostability and low brightness [189].

Imaging voltage using voltage-sensitive probes gives the highest temporal and spatial resolution among the existing in vivo functional imaging techniques [183], also allowing the subthreshold measurement of membrane potentials. Moreover, a complete 2-D map of membrane potential gives a desirable choice as functional imaging compared to other imaging techniques. Despite its superior functional imaging properties, many researchers still prefer calcium imaging mainly because of the low SNR of voltage-sensitive probes. Moreover, due to its potential toxicity of VSD and requiring genetic modification for GEVI, its application to the clinic is still not reached [189].

#### 3.2.2. Plasmonic Sensor

When illuminating the light onto the metal, the light energy will be absorbed by electrons of the metal atoms and trigger the oscillation of electrons. The electron cloud oscillation at the metal surface is called a surface plasmon (SP). Matching the momentum of light illuminated with that of the SP maximizes the energy transfer from light to the electron leading to the resonating SP [190,191]. Surface plasmon resonance (SPR) phenomena have widely been used as a biosensor since its resonance condition varies depending on the refractive index changes of the medium reflecting the concentration of biomolecules near the metal surface [191,192].

The use of SPR sensors to detect neural activity was first demonstrated by Kim et al. where an intensity-based SPR sensor with a Kretschmann configuration is employed [10]. Rat sciatic nerves were excised, and optical signals indicating the refractive index unit (RIU) were monitored. After electrical stimulation of sciatic nerve ex vivo, 10^−5^ RIU changes were detected within 5 ms without averaging. The fast optical signal changes were also monitored by Zhang et al. after culturing the hippocampal neurons on the fabricated gold nanoparticles array [193]. After illuminating 850 nm laser onto the sample, the forward scattered light was measured to monitor the resonance wavelength shift. Glutamate injection, known as a chemical neural stimulation, of the hippocampal neurons elicited differential scattered signals of ~3 × 10^−3^ without averaging. Kim et al. demonstrated optical monitoring of brain activities in vivo using gold coated optical fiber sensor [194]. RIU change was monitored to account for the neural activities after electrical stimulation. Due to the low signal to noise ratio (SNR), at least averaging of 500 optical signals is required to discern the optical signals from the noise. About ~10^−4^ RIU change was monitored after the neural stimulation whose time course resembles that obtained using the electrical recording. The mechanism underlying neural recording using the SPR sensor is not fully understood, but it might have originated from the volume change and/or ion fluctuation during neural activation [19]. Even though its advantage of recording fast optical signal using the plasmonic sensor, the low SNR as obtained in vivo study should be improved for bringing it into the clinics.

## 4. Clinical Perspective of Optical Neural Interface

The ability to deliver light energy to the target in a high spatial resolution is the key feature of neural stimulation. Likewise, delivery of photons to tissue and receiving back for detection in high spatial resolution is essential for functional imaging. Here, we will introduce several methods in order to bidirectionally transfer light energy to the target tissue with a high spatial resolution by considering the optical properties of biological tissue and parameters to be taken into account when bringing them to clinical applications.

When light interacts with the tissue, it is either reflected or scattered, or absorbed. In general, biological tissues are referred to as scattering media due to the strong optical scattering properties of the tissue [195]. Meanwhile, compared to the scattering, the absorption is minimal, especially in visible and NIR light. The mean free path due to the scattering event is only 0.1 mm while elongated up to 10~100 mm for absorption [195]. The overall extinction coefficient considering scattering and absorption has indicated that the wavelength ranging from 700 nm to 1400 nm shows comparably better tissue penetration [85]. In this sense, NIR light, known as the biological optical window, is widely used in the optical neural interface (Table 1 and Table 2).

In the case of the extrinsic neural interfaces whose light-sensitive exogenous materials absorb light in a wavelength shorter than NIR light (e.g., visible light), high-order harmonics are employed to take advantage of the NIR light [197]. NIR light could penetrate deeper into the tissue compared to visible light and selectively excite the light-sensitive materials without exciting non-target tissue. Nevertheless, overall transport means the free path is limited to ~1 mm [195]. Therefore, applications of optical neural interface targeting the brain are limited to the cortical layer of the brain and targeting deeper lost its spatial resolution significantly.

The dominant tissue compartments that block light propagation are the skin and the skull [198]. To avoid the tissue barriers, a cranial window is developed by either surgically replacing the scalp and skull with a transparent material or thinning the skull [199,200]. However, surgical removal or replacing the biological tissue hinders the optical neural interface towards the clinical application. Another approach to circumvent the skin and skull before reaching the neural cell is to select the tissue that is not enclosed. One of the good candidates is the eye which receives and detects light. Light travels through the transparent cornea, the lens and the vitreous humor before encountering the first neural cell, the retinal ganglion cell conveying visual information. The retinal neuron is a good candidate for the clinical application of an optical neural interface (Table 1 and Table 2).

When it comes to clinical application, not only the performance but also the safety is the critical parameter to be considered (Table 1 and Table 2). First, the wavelength of light should be taken into account. The light wavelength shorter than UV as well as higher energy UV light is considered as ionizing ray causing the DNA damage and the genetic mutation [84]. For long-term clinical uses, light wavelength greater than that of the visible light is essential. Next, light energy deposited onto target tissue should be considered as it generates heat (Table 1). Especially for the photothermal neural stimulation, thermal tissue damage is their biggest concern [50,201]. Since intrinsic methods of neuromodulation create bulk tissue heating, exogenous materials having good photothermal conversion efficiency are used to locally heat the neuronal membrane where it is known to be the photothermal neural activation area [87]. For the femtosecond laser stimulation, the ROS is produced, which causes problems in succession such as membrane barrier dysfunction and cell death by apoptosis [202]. Inserting exogenous materials is a potential threat that brings extrinsic optical neural interfaces to clinics (Table 1 and Table 2). The exogenous material itself (e.g., voltage sensitive dyes [203]), as well as their targeting methods (e.g., genetic modification, invasive injection of exogenous materials) can harm the biological tissue.

The level of non-invasiveness is also an important factor as it determines whether surgical treatment is required or not (Table 1 and Table 2). When it comes to optical neuromodulation, implantation of optical fiber near the target is necessary as it requires high laser intensity for activation while overcoming the tissue barrier. In this sense, the application of optical neuromodulation in the retina especially building the retinal prosthesis has the great clinical potential [100]. fNIRS and EROS are the complete non-invasive optical neuroimaging technique, while others use a cranial window for brain functional imaging. Likewise, functional retinal imaging is a good target for these types to guarantee the safety issue [204,205].

Over the past several decades, efforts to build optical neural interfaces have led several techniques to clinical applications. Nevertheless, most of them are still limited to the research field. Future endeavors are expected to overcome current technical challenges and promote the development of next-generation optical neural interfaces offering various clinical applications.

## Figures and Tables

**Table 1:** Optical neural interface for controlling the neural activities.

Method	Wavelength [nm]	Optical Intensity	Safety	Human-Application
Femtosecond laser Stimulation	750–850	~70 mW	possible safety issue due to ROS	
Infrared Neural Stimulation	1844–2120	PNS: ~700 mJ/cm^2^ CNS: ~550 mJ/cm^2^	possible safety issue due to thermal tissue damage	
Optogenetics	400–700	~8–12 mW/mm^2^	safe	Vision Recovery [68]
Photoactive Molecules Based Optical Stimulation	355–500	~3.5 mW/mm^2^	possible safety issue due to potential toxicity of cage	
Nanomaterials Based Optical Stimulation	Photothermal : 500–780 Photovoltaic : 900	Photothermal : ~159 mJ/cm^2^ photovoltaic: (material dependent) -photodiode: 0.5 µW/mm^2^ -graphene: 2.1 mW/mm^2^	safe	

**Table 2:** Optical neural interface for reading out the neural activities.

Method	Spatial Resolution	Temporal Latency	Non-Invasiveness	Robustness	Human Application
EROS	~5 mm	~100 ms (fast)	source and detector placed outside of skin (NIR range)	poor	Functional cortical imaging [114,196]
fNIRS	~1 cm	~1 s (slow)	source and detector placed outside of skin (NIR range)	robust	Various human application including psychology, linguistics, medical application (epilepsy, pain assessment, parkinson’s disease)
LDF	~100 µm	~1 s (slow)	requires cranial window when imaging neurovascular component (NIR range)	robust	functional imaging of brain cortex and optic nerve [146,149]
LSCI	~10 µm	~1 s (slow)	requires cranial window when imaging neurovascular component (NIR range)	robust	functional brain mapping [163]
Calcium Probe	~1 µm	~1 s (slow)	requires cranial window (Visible range)	robust	-
Voltage-sensitive Probe	~1 µm	~1 ms (fast, captures action potential)	requires cranial window (Visible range)	robust	-
Plasmonic Sensor	point measurement	~1 ms (fast, captures action potential)	Invasive requiring insertion of plasmonic material placed near the neuron (NIR range)	robust	-

## Data Availability

Data sharing not applicable.
